# Effect of Different Roughage Sources in Fermented Total Mixed Ration and Energy Intake on Meat Quality, Collagen Solubility, Troponin T Degradation, and Fatty Acids of Native Thai Cattle Longissimus Muscle

**DOI:** 10.3390/foods12183402

**Published:** 2023-09-12

**Authors:** Achara Lukkananukool, Sineenart Polyorach, Kritapon Sommart, Chanporn Chaosap

**Affiliations:** 1 Department of Animal Technology and Fishery, School of Agricultural Technology, King Mongkut’s Institute of Technology Ladkrabang, Bangkok 10520, Thailand; achara.lu@kmitl.ac.th (A.L.); sineenart.po@kmitl.ac.th (S.P.); 2 Department of Animal Science, Faculty of Agriculture, Khon Kaen University, Khon Kaen 40002, Thailand; kritapon@kku.ac.th; 3 Department of Agricultural Education, School of Industrial Education and Technology, King Mongkut’s Institute of Technology Ladkrabang, Bangkok 10520, Thailand

**Keywords:** Thai beef, aging, collagen content, shear force, protein degradation

## Abstract

The effects of roughage sources in the fermented total mixed ration (FTMR) and the level of energy intake on meat quality, collagen solubility, and troponin T degradation in longissimus thoracis (LT) muscle of native Thai cattle (NTC) were investigated. Results showed that roughage source affected fatty acid composition in the LT muscle (*p* < 0.05), as NTC fed Pakchong 1-Napier-based FTMR had higher monounsaturated fatty acid content and ω 6:ω 3 ratio. The high-energy ad libitum group had lower drip loss, lower shear force, and higher percent collagen solubility (*p* < 0.05). However, energy intake had no effect on troponin T degradation and fatty acid composition (*p* > 0.05). Longer aging of 14 days showed lower shear force values, higher collagen solubility, and troponin T degradation rate but higher cooking loss (*p* < 0.01). In conclusion, the meat quality of NTC could be improved by ad libitum feeding with NG-FTMR, as their meat had higher MUFA content, lower drip loss, lower shear force, and higher collagen solubility. In addition, the tenderness of NTC meat could be further improved by longer aging of 14 days post-mortem.

## 1. Introduction

As the demand for beef in Thailand increases with insufficient beef production, beef and beef products are increasingly imported from other countries such as Australia and New Zealand to meet beef demand [1]. Cattle raised in Thailand are native and crossbred Thai cattle. About 3.5 million head, or 56% of all cattle raised in Thailand, are native Thai cattle (NTC), which are classified as *Bos indicus* [2]. The advantages of NTC are good adaptation to harsh environmental conditions, resistance to ticks, parasites, and diseases, high fertility, and better utilization of low-quality feed [3]. The disadvantages of NTC are small size, low growth rate, low productivity, and tougher meat compared to *Bos taurus* crosses [3,4]. However, NTC meat may be healthier because it has lower fat and cholesterol content but higher polyunsaturated fatty acid (PUFA) content than *Bos taurus* crosses [5,6].

Considering the advantages of native Thai cattle, the productivity and meat quality of NTC need to be increased to meet consumer demand. Feed availability and feeding techniques in Thai cattle production are usually based on rice straw or other low-quality crop by-products, which are the main cause of low productivity of cattle fattening in tropical developing countries [3]. Feed intake, digestion, and energy supply of cattle are affected by the poor quality of the feed, which in turn reduces cattle performance [7,8,9,10]. Concentrate-fed cattle have higher palatability than grass-fed cattle, more intramuscular fat consisting of monounsaturated fatty acids (MUFA), and less connective tissue, which can improve flavor, juiciness, and tenderness [11]. Roughage is an essential component of ruminant diets because it provides the fiber needed for healthy rumen function. However, the disadvantage of rice straw as a roughage source for ruminants is its low dry matter intake, low protein content, and high silica and lignin content, which contribute to poor nutrient digestibility and utilization [12]. The hybrid grass Pakchong 1-Napier grass originated in Thailand from a cross between pearl millet (*Pennisetum glaucum*) and common Napier grass (*Pennisetum purpureum*) [13]. The hybrids of Napier grass and pearl millet play an important role in producing high-quality forage with lower water requirements for livestock production in tropical regions [14]. Since several studies have found that a feeding strategy can improve the performance, productivity, and meat quality of cattle [15,16,17], fermented total mixed ration (FTMR) is being considered for feeding native cattle in Thailand. FTMR is a complete feed composed of concentrates and roughages, formulated to meet the nutritional requirements of cattle and fermented to improve nutrient utilization, feed intake, digestibility, and aerobic stability [10,18,19,20,21].

Post-mortem aging in a vacuum pack or wet aging has been shown to improve meat palatability [22]. The appearance of polypeptide 30 kDa, the degradation product of troponin T, suggests that post-mortem proteolysis of myofibrillar proteins contributes to improved tenderness during aging [23,24,25]. Proteolytic enzymes also destroy and degrade muscle intramuscular connective tissue structure (IMCT) during prolonged aging [26]. The combination of high collagen concentration and limited collagen solubility makes meat tough [27,28]. The fatty acid profile of meat is important for health. Saturated fatty acids (SFA) can lead to health problems, so monounsaturated fatty acids (MUFA), which have more health benefits, are preferable [29]. Different saturated fatty acids have different biological effects, which explains their function in cardiovascular diseases [30].

This study was a continuation of the research conducted by Sommart et al. [31], which investigated the chemical composition and fermentation quality of FTMR, growth performance, digestibility of feed treatments, rumen fermentation characteristics, carcass quality, and meat physicochemical properties, and production costs of NTCs fed different roughage sources in FTMR consisting of rice straw (RS-FTMR) or Pakchong 1-Napier grass (NG-FTMR) and fed different energy intake levels; 1.5 × Metabolizable energy requirement for maintenance (1.5M) and ad libitum (ad lib). Therefore, the objective of this study was to further investigate the effects of feeding native Thai cattle different roughage sources in FTMR and energy intake on (1) meat quality, collagen solubility, and troponin T degradation of 2- or 14-day aged LT muscle and (2) meat color, drip loss, and fatty acid composition of 2-day aged LT muscle.

## 2. Materials and Methods

### 2.1. Animal Ethics

The 20 NTC bulls that were part of a government project funded by the Thailand Research Fund (project code RDG5820025) provided the LT muscle samples used for this study. The cattle were raised in the Khon Kaen province of Thailand at the Khon Kaen University Farm Research Station [31]. All experimental methods were conducted in accordance with the Animal Care and Use Committee of the Department of Livestock Development, Ministry of Agriculture and Cooperatives, Royal Thai Government animal welfare standard.

### 2.2. Experimental FTMR, Animals, Design, and Data Collection

Twenty NTC bulls were randomly assigned to 2 × 2 factorial experiments with two different roughage sources in FTMR, Pakchong 1-Napier grass and rice straw, and two different energy intake levels, 1.5 × Metabolizable energy requirement for maintenance (1.5M) and ad libitum. The bulls’ average age and initial body weight were 29.5 ± 0.62 months and 134 ± 9.51 kg, respectively. The experimental diet was formulated to meet the nutrient requirements of beef cattle [32] and to produce a total mixed ration (TMR) silage [21]. The ingredients and analyzed chemical composition of the feed are shown in Table S1 [31]. Bulls were randomly assigned to one of five blocks according to their initial body weight. Within each block, animals were assigned to one of four feed treatments and housed in individual pens (3 m × 4 m) with free access to daily feed and drinking water for 450 days of feeding trials. The differing energy intake levels were controlled by restricted feeding based on body weight for metabolizable energy requirement for the maintenance (1.5M) group and offered on ad libitum for the ad libitum energy intake group. After rearing, bulls were slaughtered in a slaughterhouse using commercial methods after 12 h of feed deprivation. After slaughter, carcasses were split lengthwise and stored at 2 ± 2 °C for 24 h before transfer to the Meat Science and Technology Laboratory at King Mongkut’s Institute of Technology Ladkrabang, Bangkok, Thailand. At 2 days post-mortem, the carcasses were dissected, and the longissimus thoracis (LT) muscles were removed from the left side of each carcass. After all visible fat was removed, each LT muscle was divided into one 1.5 cm thick and two 5 cm thick portions. The 1.5 cm thick portion was vacuum packed and stored at −80 °C for further analysis of fatty acid composition. Each of the 5 cm thick portions was individually vacuum packed and stored at 1 °C for 2 or 14 days. To analyze meat quality, each 5 cm thick muscle portion was removed from a barrier bag at each post-mortem aging time point, and pH was measured. The muscle was then cut into 2 and 3 cm thick steaks, individually vacuumed in a barrier bag, and frozen at −20 °C. Each 2 cm thick steak was used for analysis of collagen content and troponin T degradation, while the 3 cm thick steak was used for measurement of cooking loss and Warner–Bratzler shear force (WBSF).

### 2.3. Meat Quality Analysis

A pH meter with a spear-tip glass electrode (SevenGo, Mettler-Toledo International Inc., Greifensee, Switzerland) was used to measure muscle pH in duplicate. After exposure to oxygen for 45 min at 25 °C, the CIE L*, a*, and b* color values were measured in triplicate using a portable spectrophotometer (MiniScan EZ, illuminance D65, 10° observer, Hunter Associates Laboratory Inc., Reston, VA, USA) with an aperture of 2.54 cm diameter.

For drip loss, muscle samples were weighed, hung in a closed high-density polyethylene bag, stored at 2 ± 2 °C for 48 h, and re-weighed. Drip loss was reported as a percentage and calculated as follows:

% Drip loss = [(original weight − after 48 h weight)/(original weight)] × 100

For cook loss, each 3 cm thick slice was weighed and then placed in a high-density polyethylene bag, sealed with heat, and cooked in a water bath at 80 °C until the internal temperature reached 70 °C. After the cooked samples were cooled to room temperature, they were removed from the bag, re-weighed, and cook loss was reported as a percentage and calculated as follows:

% Cook loss = [(raw weight − cooked weight)/(raw weight)] × 100

For WBSF measurements, each cooked sample was cut into eight cubes of 1.3 × 1.3 × 3 cm^3^ across and along the muscle fibers. The texture analyzer (EZ-SX, Shimadzu, Kyoto, Japan) with a 50 kg load cell and a crosshead speed of 50 mm/min was used to cut each cube so that the muscle fibers were severed. WBSF values were obtained from the average number of eight cubes.

### 2.4. Collagen Analysis

Collagen analysis was performed as described by Chaosap et al. [33]. Briefly, the muscle sample was homogenized with Ringer’s solution at 77 °C for 70 min and then centrifuged at 2500× *g* for 10 min. Hydrolysis of the supernatant and sediment in 12 N HCl and 6 N HCl at 110 °C, respectively, was used to determine soluble and insoluble collagen. The concentration of hydroxyproline was calculated by measuring the absorbance of the hydrolysate at 550 nm and comparing it with a standard curve. The collagen content was calculated by multiplying the hydroxyproline content by 7.25.

### 2.5. Troponin T Degradation Analysis

Troponin T degradation was analyzed according to the method described by Chaosap et al. [33]. Briefly, the muscle sample was homogenized in an extraction buffer (50 mM Tris/HCl pH 7.5, 5 mM EDTA, with protease inhibitors) and then an equal volume of a sample buffer (125 mM Tris-HCl pH 6.8, 4% (*w*/*v*) sodium dodecyl sulfate, 0.1 M dithiothreitol, 20% (*v*/*v*) glycerol, 0.01% (*w*/*v*) bromophenol blue) was added to the homogenate. The mixture was boiled for 5 min before being applied to a pre-made 10% stain-free gel containing 5% stacking gel. A 10–250 kDa protein marker was used to identify the proteins. Using anti-troponin T (T6277, Sigma–Aldrich, Darmstadt, Germany) as the primary antibody and anti-mouse (A9044, Sigma–Aldrich, Darmstadt, Germany) as the secondary antibody, immunological detection was performed after electrophoresis gel separation of the proteins on polyvinylidene difluoride membranes. A chromogenic substrate was used to identify membrane proteins. Quantity One Multi Analyst Imaging Software Version 4.5 (Bio-Rad, Hercules, CA, USA) was used to determine the intensity of visually detectable bands.

### 2.6. Fatty Acids Analysis

Fatty acid analysis was performed as described by Chaosap et al. [34]. A total of 5 g of the pulverized muscle sample was weighed into a 500 mL Erlenmeyer flask and homogenized in 30 mL of a mixture of chloroform and methanol (2:1, *v*/*v*) at 12,000 rpm for 2 min. Then, the homogenized sample was placed in a separatory funnel; 10 mL of chloroform, 10 mL of deionized water, and 25 mL of 0.58% sodium chloride were added, mixed thoroughly, and allowed to stand for 12 h at room temperature. The lower chloroform phase was collected and evaporated using a rotary evaporator to obtain the extracted lipid. The chloroform was added to the extracted lipid to adjust it to a final volume of 10 mL. To prepare the fatty acid methyl esters (FAMEs), methyl nonadecanoate (C19:0) was added as an internal standard to 2 mL of the lipid extracted in chloroform. Methyl nonadecanoate was used as an internal standard during extraction. FAMEs were analyzed by gas chromatography (7890B, Agilent, Santa Clara, CA, USA) using a fused silica capillary column (100 m 0.25 mm 0.2 m film thickness, SPTM-2560, Supelco, Bellwood, PA, USA). The following are the gas chromatography conditions: injected temperature, 240 °C; detector temperature, 260 °C; carrier gas, He; split ratio, 10:1; temperature program, initial temperature 60 °C, followed by an increase of 20 °C/min to 170 °C, 5 °C/min to 220 °C, then, 2 °C/min to 240 °C. The peaks of the FAMEs were identified and quantified by comparing the retention times of the components FAME C4-C24 with the standard.

### 2.7. Statistical Analysis

Data were analyzed with a general linear model using Proc GLM. The least squares means were separated using the PDIFF option. Values of *p* < 0.05 were considered statistically significant. Statistical analysis was performed using SAS Institute Inc. in Cary, NC, USA. Since the interaction term is not statistically significant, it was removed from the model, and the following experimental models were used for meat color, drip loss, and fatty acid composition: Yijk = µ + bi + Rj + Ek + €ijk, where Yij was the dependent variable, µ was the overall mean, bi was the block effect of initial body weight (i = 1, …, 5), Rj was the fixed effect of roughage source (j = 1, 2), Ek was the energy level (k = 1, 2), and €ijk was the residual error. For pH, cooking loss, shear force, collagen solubility, and troponin T degradation, the experimental model was used according to the following equation: Yijkl = µ + bi + Rj + Ek + Al + €ijkl, where Yijkl was the dependent variable, µ was the overall mean, bi was the block effect of initial body weight (i = 1, …, 5), Rj was the fixed effect of roughage source (j = 1, 2), Ek was the energy level (k = 1, 2), Al was the fixed effect of aging time (l = 1, 2), and €ijkl was the residual error.

## 3. Results

In this study, the effects of roughage sources and energy intake on the meat characteristics of beef cattle were investigated. No significant effect of interaction between roughage source and energy intake was found. Therefore, only the effect of the main effect was present. Information on carcass characteristics of cattle used in this study was reported by Polyorach et al. [35]. For roughage source, slaughter weight, hot carcass weight, and hot carcass percentages were 351.7 and 271.3 kg, 174.6 and 169.9 kg, and 59.5 and 57.1% for RS and NG groups, respectively. For the effects of energy intake, the slaughter weight, hot carcass weight, and hot carcass percentage were 318.5 and 304.5 kg, 173.1 and 171.3 kg, and 58.8 and 57.8% for the 1.5M and ad lib groups, respectively. Furthermore, the slaughter age of the animals ranged from 43.3 to 43.6 months. From the study of Polyorach et al. [35], group RS had significantly higher slaughter weight than group NG, without a significant effect on hot carcass weight and hot carcass percentage. A possible explanation for the higher slaughter weight of the RS group could be the higher average daily gain and feed intake, as reported by Sommart et al. [31].

### 3.1. Meat Quality

There was no effect of roughage source or energy intake on the color of the NTC LT muscle (*p* > 0.05), as shown in Table 1. Drip loss was not affected by the roughage source but was affected by the level of energy intake, as drip loss was lower in the NTC LT muscle of the ad lib group than in the 1.5M group (*p* < 0.05). The lower the drip loss, the better the meat quality with high energy intake.

There were no effects of roughage source on pH and shear force (*p* > 0.05), as shown in Table 2. However, the tenderness of NTC beef could be improved by high energy intake, as the shear force of the LT muscle from the ad lib group had a lower shear force value than that of the 1.5M group (*p* < 0.05). The longer aging time may improve the tenderness of NTC beef, as the shear force of 14-day-aged beef was lower than that of 2-day-aged beef (*p* < 0.01). However, the longer aging time caused more cooking losses (*p* < 0.01).

### 3.2. Collagen Solubility

Roughage source, level of energy intake, and aging time did not affect soluble, insoluble, or total collagen content (*p* > 0.05), as shown in Table 3. However, insoluble collagen content tended to be higher in the NTC LT muscle of the ad lib group than in the 1.5M group (*p* < 0.1). Energy intake and aging time affected the percentage of collagen solubility (*p* < 0.05), as high energy intake and longer aging time resulted in higher collagen solubility in the NTC LT muscle. The higher collagen solubility at high energy intake and longer aging time could improve the tenderness of NTC beef. In addition, roughage source tended to affect the percentage of collagen solubility (*p* < 0.1), as the LT muscle from the group fed NG-FTMR tended to have a higher percentage of collagen solubility (*p* < 0.1).

### 3.3. Troponin T Degradation

**Figure 1:**
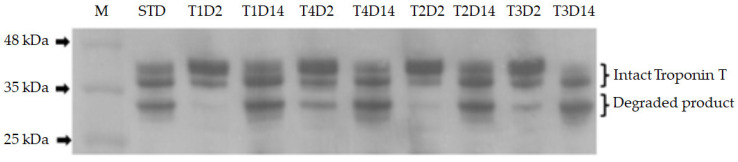
Representative Western blot of whole muscle protein extracted from longissimus thoracis of native Thai cattle aged for either 2 or 14 days subjected to different roughage sources (rice straw, Pakchong 1-Napier grass) and energy levels (1.5M, ad lib); T1 = rice straw and 1.5M, T2 = rice straw and ad lib, T3 = Pakchong 1-Napier grass and 1.5M, T4 = Pakchong 1-Napier grass and ad lib; D2 = aging for 2 days, D14 = aging for 14 days; M = protein standard 25–250 kDa; STD = represent a standard extract from one sample aged for 14 days used for interblot comparisons.

Immunoreactive bands 37 and 30 kDa were measured for the intact and degraded forms of troponin T, respectively (Figure 1). There were no significant effects (*p* > 0.05) of roughage source, energy intake, and aging time on the relative band intensities of intact troponin T (Table 4). Similarly, the relative band intensities of the degraded product and the percentage of the degraded product of troponin T were not affected by either roughage source or energy intake (*p* > 0.05). However, the relative band intensities of degraded troponin T and the percentage of degradation product increased significantly with increasing aging time (*p* < 0.01), indicating an improvement in the tenderness of NTC beef during aging.

### 3.4. Fatty Acid Composition

The effects of roughage source in FTMR and level of energy intake on fatty acid composition in the NTC LT muscle are shown in Table 5. Roughage sources had a significant effect on some fatty acid compositions, as the LT muscle from NTC fed NG-FTMR had higher levels of myristic acid (C14:0), stearic acid (C18:0), arachidic acid (C20:0), oleic acid (C18:1n9c), arachidonic acid (C20:4n6), MUFA, and also a higher ω 6:ω 3 ratio, but lower levels of docosahexaenoic acid (C22:6n3) and ω 3 than NTC fed RS-FTMR (*p* < 0.05). Energy intake did not affect the fatty acid composition, except that LT muscles of NTC fed ad libitum tended to have higher levels of myristic acid and myristoleic acid than NTC fed 1.5M (*p* < 0.1).

## 4. Discussion

### 4.1. Meat Quality

Color is the most influential of the many quality factors of fresh meat on purchase decisions. A typical indicator of fresh meat is a bright cherry red color [36,37]. Many factors influence meat color, including pH, muscle type, age, animal species, and feed source [36]. In the current study, neither roughage source in FTMR nor energy intake had any effect on the meat color of the NTC LT muscle. However, O’Sullivan et al. [38] found that the type of roughage influenced meat color, as cattle fed maize silage had poorer color stability than those fed grass silage. Keller et al. [16] found that the a* value of the longissimus thoracis was higher in bulls fed grass silage, corn-cob mix, and concentrate (ratio of 500:300:200) than in cattle fed little grass silage and mostly maize silage and concentrate (ratio of 100:600:300). Ku et al. [39] found no significant effect of different forage proportions in TMR on meat color of Hanwoo sirloins. In contrast, Kang et al. [40] found a significant increase in a* and a trend toward an increase in L* and b* in the longissimus dorsi (LD) muscle of Hanwoo steers fed high-energy diets compared with the group fed a basal diet. The explanation for the improvement in meat color by feeding a high-energy diet could be influenced by muscle metabolism, intramuscular fat, and muscle fiber properties [40,41]. In the current study, meat color was not affected by energy intake, possibly due to differences in breed, feeding, timing of meat color measurement, or sampling, because color in the current study was measured on chilled meat two days post-mortem, whereas in Kang et al. [40] it was measured after thawing of muscle samples.

Drip loss is an important quality characteristic of fresh meat. It can lead to economic losses because the meat tissue loses weight during storage. The fluid that leaks from fresh meat is referred to as drip loss or purge loss. This definition refers to the liquid leaving the meat without any mechanical force other than gravity [42]. The roughage source had no effect on drip loss in this study, which is consistent with the results of Keller et al. [16], who found no effect of different roughage source ratios–grass silage, maize silage, and corn-cob mix-on drip loss of LT muscles of Limousin-sired bulls, which ranged from 1.05% to 1.35%. He et al. [43] also found no significant difference in drip loss of striploin from cattle fed corn silage or corn stalk silage in total mixed rations. In this study, the lower drip loss in the high-energy diet group could be related to the numerically higher total fatty acids in this group compared to the 1.5M group. According to Wang et al. [15], high energy intake can alter rumen fermentation by increasing amylolytic bacteria and decreasing cellulolytic bacteria, which in turn leads to an increase in propionate concentration and a decrease in acetate concentration, further increasing intramuscular fat because propionate can provide a substrate for intramuscular lipogenesis. The increased intramuscular fat may improve water retention by reducing drip loss and cooking loss [15,44,45].

Changes in muscle pH are important in the evaluation of meat quality because they correlate with traits such as water-holding capacity and tenderness [46]. In this study, muscle pH was not affected by roughage source, energy intake, or aging time. Ku et al. [39] found no effect of varying roughage percentages in the total mixed ratio on the muscle pH of Hanwoo steers. Keller et al. [16] also reported no effect of different ratios of roughage sources on the muscle pH of Limousin-sired bulls. The level of energy intake had no effect on muscle pH in this study. This is in agreement with Wang et al. [15], who found no effect of energy intake on the muscle pH of Holstein–Friesian bulls. In contrast to our study, Li et al. [47] reported that the longissimus muscle of steers fed high-energy diets had a lower ultimate pH than that of steers fed low-energy diets. They explained that a high-energy diet could result in higher glycogen availability and lower muscle pH. Energy intake may affect muscle pH differently depending on the breed of cattle, age, or diet. Sommart et al. [31] found that growth indices of NTC, especially average daily gain (ADG), did not improve with different energy intakes, possibly due to reaching mature body weight in the finishing phase. In the current study, which was the continuation study of Sommart et al. [31], the lower nutrient utilization of NTC may have resulted in comparable muscle glycogen levels at different energy intakes, and muscle pH did not differ.

Cooking loss is the loss of water when meat is cooked. It indicates the ability of meat to retain water during cooking at high temperatures and is negatively correlated with meat juiciness [48]. High cooking loss is an important economic indicator for the meat-processing sector because it reduces meat yield and changes the appearance of meat [49]. In this study, roughage source tended to affect cooking loss, as LT muscles from the NG-FTMR group had higher cooking loss than those from the RS-FTMR group. Keller et al. [16] found no significant effect of roughage source on the cooking loss of longissimus thoracis et lumborum of Limousin-sired bulls. He et al. [43] also found no significant difference in cooking loss of striploin from cattle fed either corn silage or corn stalk silage in total mixed rations. Energy intake did not affect cooking loss in this study, which is consistent with the results of Wang et al. [15], who found no significant differences in cooking loss of LD of Holstein–Friesian bulls when fed different levels of energy, which ranged from 28.7 to 31.5%, higher than in this study (23%). The difference could be due to cattle breed, feed, or cooking method. In the present study, cooking loss was found to increase with aging time. This is in agreement with the results of Vaskoska et al. [50], who found a 3% higher cooking loss in beef aged for 14 days than in beef not aged. Post-mortem proteolysis, presumably due to denaturation of muscle proteins and degradation of muscle proteins, particularly troponin T, may be responsible for a reduction in the water-holding capacity of aged beef, resulting in increased cooking loss [23,51]. In addition, the ability of aged beef to remove water during cooking has been shown to increase, with water likely being transferred from the intracellular to the extracellular compartment.

The Warner–Bratzler shear force (WBSF) test, in which a weight-driven mechanical blade is cut through a meat sample, can be used to objectively assess the tenderness of meat. The lower the WBSF values, the more tender the meat. Martinez et al. [52] reported that beef cuts with a WBSF value of 2.8 kg (28 N) or less could be considered moderately tender as defined by a descriptive sensory evaluation, and a WBSF value between 3.0 and 3.2 kg (30 and 32 N) would indicate slightly tender beef. Beef with WBSF values of 4.0 kg (40 N) or higher was classified as moderately tough or tougher. In this study, shear force was not affected by roughage source. This is in agreement with He et al. [43], who found no effect of roughage source, corn silage, or corn stalk silage in TMR on steer sirloin shear force. According to Aberly et al. [53], cattle fed high-energy diets had lower shear force values and a higher muscle fragmentation index than cattle fed low-energy diets. Chaosap et al. [54] reported that NTC fed more cassava pulp in FTMR with higher metabolizable energy had significantly lower shear force values, and it was suggested that the lower WBSF value of NTC fed high-energy FTMR may be due to greater marbling compared to cattle fed low-energy FTMR. Wang et al. [15] found that high-energy diets with less structural carbohydrates increased amylolytic rumen bacteria but decreased cellulolytic bacteria in Holstein–Friesian bulls. As a result, propionate concentration increased while acetate concentration decreased. This changed the amount of glucose in the blood, and more carbon was available for intramuscular lipogenesis [15]. However, Wang et al. [15] reported higher intramuscular fat in the high-energy group but found no significant difference in shear force in cattle fed different energy levels. As expected, 14-day-aged beef had lower WBSF than 2-day-aged beef in the present study. The explanation for this could be post-mortem proteolysis by endogenous enzymes, especially calpains, which cause protein degradation, leading to a lower shear force value in longer-aged meat [23,24].

### 4.2. Collagen Solubility

In meat, collagen is an essential component of connective tissue. The toughness of meat can be increased by higher collagen content, lower collagen solubility, or stronger structural cross-linking of collagen [27,28]. In the present study, the LT muscles of cattle fed Napier grass as a roughage source in FTMR tended to have higher collagen solubility than those of cattle fed rice straw as a roughage source in FTMR. LT muscles from the high-energy ad lib group had a higher percentage of collagen solubility than those from the 1.5M group. This is consistent with Aberle et al. [53], who found that the longissimus muscle from cattle fed high-energy diets had higher collagen solubility than that of cattle fed low-energy diets. According to the results of Silva et al. [55], the longissimus muscle of gazing bulls fed an energy supplement had higher collagen solubility compared to cattle fed low-energy grass, possibly due to higher new collagen formation, which is less cross-linked. In this study, there was no effect of aging time on collagen content. However, the longer aging of 14 days had a higher percentage of collagen solubility than the aging of 2 days. Fan et al. [48] reported a similar result: aging time had no effect on collagen content. Mikołajczak et al. [56] found that collagen solubility increased during aging from 45 min to 21 days. They found that the highest collagen solubility after 3 days of aging was between 10.99% and 12.82%, while after 21 days of aging, it was between 11.05% and 16.42%. Proteoglycans (PGs), along with collagen, are among the protein elements of intramuscular connective tissue (IMCT) that stabilize the structure of collagen myofibrils and IMCT. The solubility of collagen could be due to the degradation of PGs and the weakening of the connections between collagen fibrils, resulting in increased tenderness of the meat. The degradation of IMCT during aging is related to the activity of the primary enzymes, metalloproteinases (MMPs) [28,57].

### 4.3. Troponin T Degradation

Measurement of troponin T degradation is commonly used as an indicator of myofibrillar protein degradation during post-mortem aging. Several studies reported the relationship between the degree of troponin T degradation and meat tenderness [23,33,58]. In addition, Steen et al. [59] reported that shear force correlated positively with intact troponin T and negatively with its degradation product. In the present study, neither roughage source nor energy content affected troponin T degradation. Only aging time affected the post-mortem proteolysis of troponin T, which is in agreement with [23,33,58]. Blank et al. [60] and McDonagh et al. [61] noted that the relationship between high growth performance and high calpastatin activity, the inhibitor of endogenous calpain enzymes, could contribute to higher productivity, as lower protein degradation should increase net muscle gain. Unfortunately, lower protein breakdown during post-mortem aging results in tougher meat. The lack of effect of energy intake on troponin T degradation observed in this study may be due to the similar growth performance of the same cattle examined in the study by [31].

### 4.4. Fatty Acid Composition

In this study, roughage sources altered the fatty acid profile. The differences in fatty acid composition in LT muscles relative to the roughage sources in FTMR could be explained by the differences in nutrient intake. As mentioned in Table S1, Pakchong 1-Napier grass had higher fat and protein content than rice straw, and NG-FTMR also had slightly higher organic matter, fat, and protein content than RS-FTMR. In addition, Sommart et al. [31], who provided the LT muscle samples used in this study, reported that NG-FTMR had slightly higher organic matter (93.4 vs. 91.8% DM) and protein (16.1% vs. 14.1%) content than RS-FTMR after 21 days of ensiling. They also reported that NG-FTMR had higher lactic acid (51.4 vs. 43.4 g/kg DM), acetic acid (39.0 vs. 27.1 g/kg DM), and propionic acid (3.3 vs. 1.9 g/kg DM) content after 21 days of ensiling compared to RS-FTMR. The higher fermentation forage fat and carbohydrate, such as ruminal acetic acid as a precursor of fatty acid synthesis from NG-FTMR, could explain the higher levels of myristic acid, stearic acid, arachidic acid, oleic acid, arachidonic acid, and MUFA in the LT muscle of cattle fed NG-FTMR compared to cattle fed RS-FTMR. According to Randby et al. [62], the chemical composition of silage—crude protein, crude fat, water-soluble carbohydrates, and metabolizable energy—decreased with increasing plant maturity. They also found that oleic acid and some of the MUFA in intramuscular fat decreased with increasing plant maturity. De Smet et al. [63] found significant effects of dietary energy content on fatty acid composition in LT muscles of Belgian Blue bulls, as they found that high dietary energy content had higher levels of C14:0, C16:0, C16:1, C18:1, and MUFA, but lower levels of C18:2, C20:4, and PUFA than low dietary energy content. However, the difference in energy intake did not affect fatty acid composition in the present study, possibly due to the limited nutrient utilization of NTC, as shown by the lack of difference in average daily gain and weight gain when comparing the different energy intakes, as mentioned in the report by [31]. Kang et al. [40] found that most fatty acid compositions did not show significant differences between the different energy diets, except for stearic acid, which was higher in the LD muscles of Hanwoo steers fed high-energy diets. The fatty acid composition may vary among studies depending on cattle breed, diet, sex, and age.

The three most abundant fatty acids in the LT muscle of NTC in the current study were oleic acid, palmitic acid, and stearic acid, which is consistent with the results of [54,64,65]. The fatty acid composition in the bovine muscle depends on the fatty acids in the diet and the activity of rumen microorganisms that convert unsaturated fatty acids to saturated fatty acids [66]. MUFAs and PUFAs are generally considered to be beneficial to health by increasing high-density lipoprotein (HDL) cholesterol and reducing the risk of cardiovascular disease, whereas SFAs are considered to be detrimental to human health [29]. However, high levels of PUFAs in meat can cause oxidative rancidity, which affects the taste, shelf life, and overall quality of the meat [67]. Oleic acid, found in grain-fed beef, is an important source of MUFAs for humans [68]. Omega-3 polyunsaturated fatty acids (PUFAs) such as α-linolenic acid (ALA; 18:3 ω-3), eicosapentaenoic acid (EPA; 20:5 ω-3), and docosahexaenoic acid (DHA; 22:6 ω-3) have many health benefits, for example, in heart disease, diabetes, cancer, depression, and other mental health conditions [69]. Omega-3 PUFAs lower plasma triglycerides by decreasing hepatic synthesis of very low-density lipoproteins, triglyceride synthesis, and de novo lipogenesis [70]. For high triglycerides, the American Heart Association recommends more than 3 g EPA and DHA per day as treatment or as an adjunct to lipid-lowering medications [71]. The recommended ω-6:ω-3 ratio varies from 1:1 to 4:1 [72]. The diet with the lowest ω-3:ω-6 ratio (1:1) resulted in the least atherosclerosis formation, and the severity of atherosclerosis increased with increasing ω-3:ω-6 ratio [73]. In this study, the LT muscle of cattle fed RS-FTMR had a higher DHA level with a lower ω-3:ω-6 ratio compared with the NG-FTMR group. Therefore, the meat from the RS-FTMR group appears to have greater human health benefits than that from the NG-FTMR group. However, the greater amount of MUFAs in the LT muscles of the NG-FTMR group may play a more important role in the health benefits to consumers than the lower amount of MUFAs in the LT muscles of the RS-FTMR group.

## 5. Conclusions

In conclusion, the meat quality of NTC could be improved by ad libitum feeding with NG-FTMR because their meat could have higher MUFA content, lower drip loss, lower shear force, and higher collagen solubility. In addition, the longer aging of 14 days post-mortem could further improve the tenderness of NTC meat through higher collagen solubility and troponin T degradation rate. However, the higher cooking loss could be considered a disadvantage of the longer aging time.

## Figures and Tables

**Table 1:** Effect of roughage source (R) in a fermented total mixed ration and level of energy intake (E) on meat color and drip loss of longissimus thoracis of native Thai cattle aged for 2 days.

Trait	Roughage (R)		Energy Intake (E)		RMSE	*p* Value	
	NG	RS	1.5M	ad lib		R	E
L*	36.93	35.44	35.86	36.52	2.80	0.287	0.622
a*	16.72	17.05	17.01	16.76	1.75	0.691	0.763
b*	9.11	10.17	9.96	9.32	2.26	0.335	0.550
Drip loss (%)	1.87	1.59	2.09	1.37	0.47	0.285	0.017

M = metabolizable energy requirement for maintenance; NG = Pakchong 1-Napier grass; RS = rice straw.

**Table 2:** Effect of roughage source (R) in a fermented total mixed ration and level of energy intake (E) on meat quality of longissimus thoracis of native Thai cattle aged for either 2 or 14 days.

Trait	Roughage (R)		Energy Intake (E)		Aging (A)		RMSE	*p* Value		
	NG	RS	1.5M	ad lib	2 days	14 days		R	E	A
pH	5.42	5.43	5.44	5.41	5.44	5.39	0.10	0.831	0.416	0.140
Cooking loss (%)	23.32	21.49	22.33	22.48	19.10	25.61	3.02	0.076	0.884	<0.0001
Shear force (kg)	7.96	7.65	8.39	7.21	9.33	6.28	1.65	0.579	0.037	<0.0001

M = metabolizable energy requirement for maintenance; NG = Pakchong 1-Napier grass; RS = rice straw.

**Table 3:** Effect of roughage source (R) in a fermented total mixed ration and level of energy intake (E) on soluble, insoluble, and total collagen contents (mg/g wet weight) and collagen solubility (%) of longissimus thoracis of native Thai cattle aged for either 2 or 14 days.

Collagen	Roughage (R)		Energy Intake (E)		Aging (A)		RMSE	*p* Value		
	NG	RS	1.5M	ad lib	2 days	14 days		R	E	A
Soluble	0.22	0.21	0.21	0.23	0.20	0.24	0.09	0.710	0.488	0.176
Insoluble	2.91	3.10	3.23	2.78	3.10	2.91	0.77	0.462	0.096	0.478
Total	3.13	3.32	3.44	3.01	3.29	3.15	0.84	0.523	0.141	0.616
Solubility (%)	7.39	6.14	5.86	7.67	5.93	7.59	2.06	0.084	0.015	0.022

M = metabolizable energy requirement for maintenance; NG = Pakchong 1-Napier grass; RS = rice straw.

**Table 4:** Effect of roughage source (R) in a fermented total mixed ration and level of energy intake (E) on troponin T degradation of longissimus thoracis of native Thai cattle aged for either 2 or 14 days.

Troponin T	Roughage (R)		Energy Intake (E)		Aging (A)		RMSE	*p* Value		
	NG	RS	1.5M	ad lib	2 days	14 days		R	E	A
Intact ^1^	0.07	0.06	0.06	0.07	0.06	0.06	0.02	0.297	0.171	0.774
Degraded product ^1^	0.04	0.03	0.03	0.04	0.02	0.05	0.01	0.213	0.379	<0.0001
% Degraded product ^2^	32.66	31.06	33.23	30.48	21.65	42.07	5.67	0.452	0.203	<0.0001

M = metabolizable energy requirement for maintenance; NG = Pakchong 1-Napier grass; RS = rice straw. ^1^ Values indicated are a ratio of either intact or degraded product band intensity to whole protein band density in stain-free gel. ^2^ Total band intensity of intact and degraded products of Troponin T within each sample is taken as 100%. Values indicated are a relative percentage of the signal intensity of the degraded product.

**Table 5:** Effect of roughage source (R) in a fermented total mixed ration and level of energy intake (E) on fatty acid composition (mg/100 g fresh meat) of longissimus thoracis of native Thai cattle aged for 2 days.

Trait	Roughage (R)		Energy Intake (E)		RMSE	*p* Value	
	NG	RS	1.5M	ad lib		R	E
Saturated fatty acid (SFA)							
Capric acid (C10:0)	8.18	5.89	6.46	7.61	3.50	0.247	0.553
Lauric acid (C12:0)	10.18	6.58	7.07	9.69	4.46	0.160	0.299
Myristic acid (C14:0)	184.21	163.03	135.85	181.36	42.91	0.047	0.075
Pentadecylic acid (C15:0)	15.15	8.12	10.87	12.40	4.10	0.008	0.505
Palmitic acid (C16:0)	901.45	818.50	723.33	996.62	294.45	0.608	0.114
Margaric acid (C17:0)	27.55	23.17	24.23	26.48	6.64	0.243	0.543
Stearic acid (C18:0)	385.85	282.91	327.96	340.80	59.93	0.008	0.699
Arachidic (C20:0)	2.40	1.55	1.95	2.01	0.55	0.015	0.856
Monounsaturated fatty acid (MUFA)							
Myristoleic acid (C14:1)	60.05	53.60	47.79	65.86	55.53	0.526	0.097
Pentadecenoic acid (C15:1)	5.57	4.24	4.39	5.42	1.88	0.212	0.334
Palmitoleic acid (C16:1)	184.67	161.93	158.73	187.86	34.60	0.244	0.147
Heptadecenoic acid (C17:1)	19.39	18.60	18.77	19.22	2.83	0.612	0.778
Oleic acid (C18:1n9c)	1189.62	1029.70	1103.79	1115.52	131.91	0.044	0.872
Erucic acid (C20:1n9)	4.18	7.12	7.27	4.04	6.88	0.441	0.403
Erucic acid (C22:1n9)	19.02	16.39	19.27	16.14	6.99	0.497	0.424
Nervonic acid (C24:1)	4.09	4.11	4.52	3.69	2.14	0.988	0.488
Polyunsaturated fatty acid (PUFA)							
Linoleic acid (C18:2n6t)	77.31	69.01	74.85	71.46	25.31	0.552	0.808
α-Linolenic acid (C18:3n6)	3.20	2.01	2.65	2.56	1.27	0.109	0.889
Arachidic acid (C20:2)	9.79	8.40	10.08	8.11	3.96	0.524	0.374
Arachidonic acid (C20:4n6)	4.49	2.56	3.84	3.30	1.50	0.044	0.517
Docosahexaenoic acid (C22:6n3)	10.62	16.71	13.45	13.87	4.81	0.037	0.874
SFA	1534.97	1279.74	1237.73	1576.98	362.13	0.214	0.111
MUFA	1486.60	1295.69	1364.55	1417.75	158.91	0.046	0.548
PUFA	105.41	98.79	104.89	99.30	34.98	0.730	0.773
Total fatty acid	3126.99	2674.23	2707.17	3094.04	451.74	0.088	0.141
PUFA:SFA	0.07	0.08	0.09	0.07	0.03	0.855	0.209
ω 3	10.62	16.71	13.45	13.87	4.81	0.037	0.874
ω 6	84.99	73.68	81.35	77.32	27.47	0.457	0.791
ω 6:ω 3	8.50	4.45	6.41	6.55	1.74	0.001	0.888

SFA = C10:0 + C12:0 + C14:0 + C15:0 + C16:0 + C17:0 + C18:0 + C20:0; MUFA = C14:1 + C15:1 + C16:1 + C17:1 + C18:1n9c + 20:1n9 + C22:1n9 + C24:1; PUFA = C18:2n6t + C18:3n6 + C20:2 + C20:4n6 + C22:6n3; ω 3 = C22:6n3; ω 6 = C18:2n6t + C18:3n6 + C20:4n6. M = metabolizable energy requirement for maintenance; NG = Pakchong 1-Napier grass; RS = rice straw.

## Data Availability

Data is contained within the article or supplementary material.

## Supplementary Materials

The following supporting information can be downloaded at https://www.mdpi.com/article/10.3390/foods12183402/s1, Table S1: Ingredients and chemical composition of dietary energy density treatment in native Thai cattle diet.

## References

[1] Department of Trade Negotiations Beef and Beef Products. https://api.dtn.go.th/files/v3/613b2770ef41401f0822c2dc/download.

[2] Department of Livestock Development (2020). Number of Livestock in Thailand. https://docimage.dld.go.th/FILEROOM/CABDLD_BOOKSHELF2/DRAWER26/GENERAL/DATA0000/00000082.PDF.

[3] Saithong S., Chatchawan T., Boonyanuwat K. (2011). Thai indigenous cattle production provide a sustainable alternative for the benefit of small scale farmers, healthy food, and the environment. BAHGI e-J..

[4] Sethakul J., Opatpatanakit Y., Sivapirunthep P., Intrapornudom P. Beef quality under production systems in Thailand: Preliminary Remarks. Proceedings of the 13th AAAP Animal Science Congress.

[5] Duanyai S., Duanyai S., Tanasunthonsut W., Suwannee P. (2009). Natural Beef Production.

[6] Somboonpanyakul P., Sethakul J., Sivapirunthep P. (2009). Nutritional value of beef. Value of Thai Beef.

[7] Chaokaur A., Nishida T., Phaowphaisal I., Sommart K. (2015). Effects of feeding level on methane emissions and energy utilization of Brahman cattle in the tropics. Agric. Ecosyst. Environ..

[8] Ogino A., Sommart K., Subepang S., Mitsumori M., Hayashi K., Yamashita T., Tanaka Y. (2016). Environmental impacts of extensive and intensive beef production systems in Thailand evaluated by life cycle assessment. J. Clean. Prod..

[9] Kongphitee K., Sommart K., Phonbumrung T., Gunha T., Suzuki T. (2018). Feed intake, digestibility and energy partitioning in beef cattle fed diets with cassava pulp instead of rice straw. Asian-Australas. J. Anim. Sci..

[10] Subepang S., Suzuki T., Phonbumrung T., Sommart K. (2019). Enteric methane emissions, energy partitioning, and energetic efficiency of zebu beef cattle fed total mixed ration silage. Asian-Australas. J. Anim. Sci..

[11] Hwang Y.-H., Joo S.-T. (2017). Fatty acid profiles, meat quality, and sensory palatability of grain-fed and grass-fed beef from Hanwoo, American, and Australian crossbred cattle. Korean J. Food Sci. Anim. Resour..

[12] Aquino D., Del Barrio A., Trach N.X., Hai N.T., Khang D.N., Toan N.T., Van Hung N. (2020). Rice straw-based fodder for ruminants. Sustainable Rice Straw Management.

[13] Kiyothong K. (2014). Manual for Planting Napier Pakchong 1.

[14] Turano B., Tiwari U.P., Jha R. (2016). Growth and nutritional evaluation of napier grass hybrids as forage for ruminants. Trop. Grassl.-Forrajes Trop..

[15] Wang H., Li H., Wu F., Qiu X., Yu Z., Niu W., He Y., Su H., Cao B. (2019). Effects of dietary energy on growth performance, rumen fermentation and bacterial community, and meat quality of Holstein-Friesians bulls slaughtered at different ages. Animals.

[16] Keller M., Kreuzer M., Reidy B., Scheurer A., Guggenbühl B., Luder M., Frank J., Giller K. (2022). Effects on performance, carcass and meat quality of replacing maize silage and concentrate by grass silage and corn-cob mix in the diet of growing bulls. Meat Sci..

[17] Zhu X., Liu B., Xiao J., Guo M., Zhao S., Hu M., Cui Y., Li D., Wang C., Ma S. (2022). Effects of different roughage diets on fattening performance, meat quality, fatty acid composition, and rumen microbe in steers. Front. Nutr..

[18] Nishino N., Harada H., Sakaguchi E. (2003). Evaluation of fermentation and aerobic stability of wet brewers’ grains ensiled alone or in combination with various feeds as a total mixed ration. J. Sci. Food Agric..

[19] Li Y., Wang F., Nishino N. (2016). Lactic acid bacteria in total mixed ration silage containing soybean curd residue: Their isolation, identification and ability to inhibit aerobic deterioration. Asian-Australas. J. Anim. Sci..

[20] Gunha T., Kongphitee K., Sommart K. Feed intake, digestibility, growth performances and eating behavior of native Thai beef cattle fed diets differing in energy density using cassava pulp with rice straw. Proceedings of the 1st International Conference on Tropical Animal Science and Production.

[21] Kongphitee K., Sommart K. Ensilage quality, digestibility and enteric methane emission of the fermented total mixed ration in Thai native beef cattle. Proceedings of the 1st International Conference on Tropical Animal Science and Production.

[22] Kim M., Choe J., Lee H.J., Yoon Y., Yoon S., Jo C. (2019). Effects of aging and aging method on physicochemical and sensory traits of different beef cuts. Food Sci. Anim. Resour..

[23] Ho C., Stromer M., Robson R. (1994). Identification of the 30 kDa polypeptide in post mortem skeletal muscle as a degradation product of troponin-T. Biochimie.

[24] Koohmaraie M., Geesink G. (2006). Contribution of postmortem muscle biochemistry to the delivery of consistent meat quality with particular focus on the calpain system. Meat Sci..

[25] Bhat Z., Morton J.D., Mason S.L., Bekhit A.E.-D.A. (2018). Role of calpain system in meat tenderness: A review. Food Sci. Hum. Wellness.

[26] Nishimura T. (2015). Role of extracellular matrix in development of skeletal muscle and postmortem aging of meat. Meat Sci..

[27] Modzelewska-Kapituła M., Nogalski Z., Kwiatkowska A. (2016). The influence of crossbreeding on collagen solubility and tenderness of Infraspinatus and Semimembranosus muscles of semi-intensively reared young bulls. Anim. Sci. J..

[28] Purslow P.P. (2014). New developments on the role of intramuscular connective tissue in meat toughness. Annu. Rev. Food Sci. Technol..

[29] Gilmore L.A., Walzem R.L., Crouse S.F., Smith D.R., Adams T.H., Vaidyanathan V., Cao X., Smith S.B. (2011). Consumption of high-oleic acid ground beef increases HDL-cholesterol concentration but both high-and low-oleic acid ground beef decrease HDL particle diameter in normocholesterolemic men. J. Nutr..

[30] Mukhopadhyay S., Goswami S., Mondal S.A., Dutta D. (2020). Dietary fat, salt, and sugar: A clinical perspective of the social catastrophe. Dietary Sugar, Salt and Fat in Human Health.

[31] Sommart K., Tangjitwattanachai N., Nitipot P., Tumdee A., Chokcharoen S. (2017). Feed Innovation and Feeding for High Quality Beef Cattle.

[32] The Working Committee of Thai Feeding Standard for Ruminant (2010). Nutrient Requirement of Beef Cattle in Indochinese Peninsula.

[33] Chaosap C., Sitthigripong R., Sivapirunthep P., Pungsuk A., Adeyemi K.D., Sazili A.Q. (2020). Myosin heavy chain isoforms expression, calpain system and quality characteristics of different muscles in goats. Food Chem..

[34] Chaosap C., Sivapirunthep P., Takeungwongtrakul S., Zulkifli R.B.M., Sazili A.Q. (2020). Effects of Zn-L-Selenomethionine on Carcass Composition, Meat Characteristics, Fatty Acid Composition, Glutathione Peroxidase Activity, and Ribonucleotide Content in Broiler Chickens. Food Sci. Anim. Resour..

[35] Polyorach S., Lukkananukool A., Sommart K., Chaosap C. Effects of fermented total mixed ration (FTMR) using rice straw and napier Pachong1 as a roughage sources on carcass quality of Thai native beef cattle. Proceedings of the 6th Meat Science Technoly Congress.

[36] Mancini R., Hunt M. (2005). Current research in meat color. Meat Sci..

[37] Suman S.P., Joseph P. (2013). Myoglobin chemistry and meat color. Annu. Rev. Food Sci. Technol..

[38] O’Sullivan A., O’Sullivan K., Galvin K., Moloney A., Troy D., Kerry J. (2002). Grass silage versus maize silage effects on retail packaged beef quality. J. Anim. Sci..

[39] Ku M.J., Mamuad L., Nam K.C., Cho Y.I., Kim S.H., Choi Y.S., Lee S.S. (2021). The effects of total mixed ration feeding with high roughage content on growth performance, carcass characteristics, and meat quality of Hanwoo steers. Food Sci. Anim. Resour..

[40] Kang D.H., Chung K.Y., Park B.H., Kim U.H., Jang S.S., Smith Z.K., Kim J. (2022). Effects of feeding high-energy diet on growth performance, blood parameters, and carcass traits in Hanwoo steers. Anim. Biosci..

[41] Hughes J.M., Clarke F.M., Purslow P.P., Warner R.D. (2020). Meat color is determined not only by chromatic heme pigments but also by the physical structure and achromatic light scattering properties of the muscle. Compr. Rev. Food Sci. Food Saf..

[42] Huff-Lonergan E. (2009). Fresh meat water-holding capacity. Improving the Sensory and Nutritional Quality of Fresh Meat.

[43] He L., Yang J., Chen W., Zhou Z., Wu H., Meng Q. (2018). Growth performance, carcass trait, meat quality and oxidative stability of beef cattle offered alternative silages in a finishing ration. Animal.

[44] Kim C.J., Lee E.S. (2003). Effects of quality grade on the chemical, physical and sensory characteristics of Hanwoo (Korean native cattle) beef. Meat Sci..

[45] Frank D., Ball A., Hughes J., Krishnamurthy R., Piyasiri U., Stark J., Watkins P., Warner R. (2016). Sensory and Flavor Chemistry Characteristics of Australian Beef: Influence of Intramuscular Fat, Feed, and Breed. J. Agric. Food Chem..

[46] Weatherly B.H., Lorenzen C.L., Savell J.W. (1998). Determining optimal aging times for beef subprimals. J. Anim. Sci..

[47] Li L., Zhu Y., Wang X., He Y., Cao B. (2014). Effects of different dietary energy and protein levels and sex on growth performance, carcass characteristics and meat quality of F1 Angus × Chinese Xiangxi yellow cattle. J. Anim. Sci. Biotechnol..

[48] Fan Y., Han Z., Arbab A.A.I., Yang Y., Yang Z. (2020). Effect of Aging Time on Meat Quality of Longissimus Dorsi from Yunling Cattle: A New Hybrid Beef Cattle. Animals.

[49] Schonfeldt H., Strydom P. (2011). Effect of age and cut on cooking loss, juiciness and flavour of South African beef. Meat Sci..

[50] Vaskoska R., Ha M., Naqvi Z.B., White J.D., Warner R.D. (2020). Muscle, Ageing and Temperature Influence the Changes in Texture, Cooking Loss and Shrinkage of Cooked Beef. Foods.

[51] Jayasooriya S.D., Torley P.J., D’Arcy B.R., Bhandari B.R. (2007). Effect of high power ultrasound and ageing on the physical properties of bovine Semitendinosus and Longissimus muscles. Meat Sci..

[52] Martinez H.A., Miller R.K., Kerth C., Wasser B.E. (2023). Prediction of beef tenderness and juiciness using consumer and descriptive sensory attributes. Meat Sci..

[53] Aberle E.D., Reeves E.S., Judge M.D., Hunsley R.E., Perry T.W. (1981). Palatability and Muscle Characteristics of Cattle with Controlled Weight Gain: Time on a High Energy Diet. J. Anim. Sci..

[54] Chaosap C., Lukkananukool A., Polyorach S., Sommart K., Sivapirunthep P., Limsupavanich R. (2022). Effects of Dietary Energy Density in a Fermented Total Mixed Ration Formulated with Different Ratios of Rice Straw and Cassava Pulp on 2- or 14-Day-Aged Meat Quality, Collagen, Fatty Acids, and Ribonucleotides of Native Thai Cattle Longissimus Muscle. Foods.

[55] Silva C., Rego O., Simões E., Rosa H. (2010). Consumption of high energy maize diets is associated with increased soluble collagen in muscle of Holstein bulls. Meat Sci..

[56] Mikołajczak B., Iwańska E., Spychaj A., Danyluk B., Montowska M., Grześ B., Banach J.K., Żywica R., Pospiech E. (2019). An analysis of the influence of various tenderising treatments on the tenderness of meat from Polish Holstein-Friesian bulls and the course of changes in collagen. Meat Sci..

[57] Purslow P.P. (2018). Contribution of collagen and connective tissue to cooked meat toughness; some paradigms reviewed. Meat Sci..

[58] Nonsee M., Lukkananukool A., Polyorach S., Sommart K., Sazili A.Q., Chaosap C. (2018). Degradation of troponin-T associated with calpain/calpastatin genes expression in native Thai beef cattle fed different levels of energy. Int. J. Agric. Technol..

[59] Steen D., Claeys E., Uytterhaegen L., De Smet S., Demeyer D. (1997). Early post-mortem conditions and the calpain/calpastatin system in relation to tenderness of double-muscled beef. Meat Sci..

[60] Blank C.P., Russell J., Lonergan S.M., Hansen S.L. (2017). Influence of feed efficiency classification and growing and finishing diet type on meat tenderness attributes of beef steers1. J. Anim. Sci..

[61] McDonagh M.B., Herd R.M., Richardson E.C., Oddy V.H., Archer J.A., Arthur P.F. (2001). Meat quality and the calpain system of feedlot steers following a single generation of divergent selection for residual feed intake. Aust. J. Exp. Agric..

[62] Randby Å., Aass L., Haug A. (2021). Fatty acid profile and intramuscular fat concentration of *Musculus longissimus thoracis* in bulls fed grass silage harvested at one of three maturity stages, either with or without concentrate supplementation. Acta Agric. Scand. Sect. A—Anim. Sci..

[63] De Smet S., Webb E., Claeys E., Uytterhaegen L., Demeyer D. (2000). Effect of dietary energy and protein levels on fatty acid composition of intramuscular fat in double-muscled Belgian Blue bulls. Meat Sci..

[64] Jaturasitha S., Norkeaw R., Vearasilp T., Wicke M., Kreuzer M. (2009). Carcass and meat quality of Thai native cattle fattened on Guinea grass (*Panicum maxima*) or Guinea grass–legume (*Stylosanthes guianensis*) pastures. Meat Sci..

[65] Chaiwang N., Jaturasitha S., Sringarm K., Wicke M., Kreuzer M. (2015). Comparison of the Fatty Acid Profiles of the Meat of Crossbreds with 75% Charolais Blood Proportion and Thai Indigenous Upland Cattle. Chiang Mai Univ. J. Nat. Sci..

[66] Wang B., Wang Y., Zuo S., Peng S., Wang Z., Zhang Y., Luo H. (2021). Untargeted and Targeted Metabolomics Profiling of Muscle Reveals Enhanced Meat Quality in Artificial Pasture Grazing Tan Lambs via Rescheduling the Rumen Bacterial Community. J. Agric. Food Chem..

[67] Wood J.D., Enser M., Fisher A.V., Nute G.R., Sheard P.R., Richardson R.I., Hughes S.I., Whittington F.M. (2008). Fat deposition, fatty acid composition and meat quality: A review. Meat Sci..

[68] Nogoy K.M., Sun B., Shin S., Lee Y., Li X., Choi S.H., Park S. (2022). Fatty Acid Composition of Grain- and Grass-Fed Beef and Their Nutritional Value and Health Implication. Food Sci. Anim. Resour..

[69] Shahidi F., Ambigaipalan P. (2018). Omega-3 Polyunsaturated Fatty Acids and Their Health Benefits. Annu. Rev. Food Sci. Technol..

[70] Jump D.B., Depner C.M., Tripathy S. (2012). Omega-3 fatty acid supplementation and cardiovascular disease. J. Lipid Res..

[71] Skulas-Ray A.C., Wilson P.W.F., Harris W.S., Brinton E.A., Kris-Etherton P.M., Richter C.K., Jacobson T.A., Engler M.B., Miller M., Robinson J.G. (2019). Omega-3 Fatty Acids for the Management of Hypertriglyceridemia: A Science Advisory From the American Heart Association. Circulation.

[72] Balić A., Vlašić D., Žužul K., Marinović B., Bukvić Mokos Z. (2020). Omega-3 Versus Omega-6 Polyunsaturated Fatty Acids in the Prevention and Treatment of Inflammatory Skin Diseases. Int. J. Mol. Sci..

[73] Wang S., Wu D., Matthan N.R., Lamon-Fava S., Lecker J.L., Lichtenstein A.H. (2009). Reduction in dietary omega-6 polyunsaturated fatty acids: Eicosapentaenoic acid plus docosahexaenoic acid ratio minimizes atherosclerotic lesion formation and inflammatory response in the LDL receptor null mouse. Atherosclerosis.

