# Supplementary material for: Effect of Different Roughage Sources in Fermented Total Mixed Ration and Energy Intake on Meat Quality, Collagen Solubility, Troponin T Degradation, and Fatty Acids of Native Thai Cattle Longissimus Muscle
Source: Foods. 2023 Sep 12;12(18):3402. doi: 10.3390/foods12183402 (PMC10527866; doi:10.3390/foods12183402)
Supplement: Supplementary file 1 [file foods-12-03402-s001.zip › foods-2589332-supplementary.pdf]

**Table S1** Ingredients and chemical composition of dietary treatment in native Thai cattle diet (Sommart et al., 2017)

| Item[1] | RS-FTMR | NG-FTMR | Pakchong 1-Napier grass | Rice straw |
|---|---|---|---|---|
| Ingredients, %DM | | | | |
| Rice straw | 20 | - | | |
| Pak Chong 1 Napier grass | - | 20 | | |
| Cassava pulp | 28 | 28 | | |
| Cassava chip | 10 | 10 | | |
| Palm meal | 25 | 25 | | |
| Soybean meal | 10 | 10 | | |
| Rice bran | 5 | 5 | | |
| Urea | 0.7 | 0.7 | | |
| Mineral[2] | 0.8 | 0.8 | | |
| Limestone | 0.5 | 0.5 | | |
| Total | 100 | 100 | | |
| Analyzed chemical composition, %DM | | | | |
| Dry matter | 39.5 | 32.6 | 18.7 | 92.8 |
| Organic matter | 91.8 | 93.2 | 88.0 | 88.0 |
| Crude protein | 14.1 | 15.8 | 8.0 | 2.7 |
| Ether extract | 5.2 | 5.5 | 2.8 | 0.9 |
| Neutral detergent fiber | 44.5 | 41.2 | 73.0 | 77.0 |
| Acid detergent fiber | 31.0 | 29.1 | 49.4 | 51.6 |
| Energy density, MJ/kg DM | | | | |
| Gross energy | 17.8 | 17.8 | 16.1 | 16.2 |
| Metabolizable energy (calculated) | 9.6 | 9.8 | 7.9 | 6.5 |
| Cost, Baht/kg FM | 3.79 | 3.67 | 1.1 | 2.3 |
| Cost, Baht/kg DM | 10.04 | 9.72 | 5.9 | 2.5 |

[1] RS-FTMR = Rice straw base fermented total mixed ration, NG-FTMR = Pak Chong 1-Napier grass base fermented total mixed ration.

[2] Chemical compositions were calcium = 164.00 g, cobalt = 0.04 g, copper = 1.00 g, iodine = 0.04 g, iron = 2.00 g, magnesium = 2.89 g, manganese = 11.00 g, phosphorus = 80.00 g, selenium = 0.03 g, sodium = 136.60 g, sulfur =19.20 g and carrier = 1,000 g
